# TRIM22 in cancer: a context-dependent E3 ligase at the interface of interferon, p53, and tumor plasticity

**DOI:** 10.3389/fcell.2026.1977247

**Published:** 2026-10-05

**Authors:** Chenjie Qiu, Liangliang Dai

**Affiliations:** 1 Department of General Surgery, Changzhou Hospital of Traditional Chinese Medicine, Changzhou, China; 2 Department of Urology, Wujin Hospital Affiliated with Jiangsu University, Changzhou, China; 3 Department of Urology, The Wujin Clinical College of Xuzhou Medical University, Changzhou, China

**Keywords:** cancer biomarker, E3 ubiquitin ligase, interferon signaling, TRIM22, tumor plasticity

## Abstract

TRIM22 is a TRIM family E3 ubiquitin ligase whose expression is strongly induced by interferons. For many years, it has been identified primarily as an antiviral effector, a role that remains well validated. A growing body of cancer-related work now forces us to expand that view considerably. Depending on tumor type, upstream signaling context, and the specific substrates available in a given cell, TRIM22 can either constrain malignancy or actively promote it. In breast, colorectal, ovarian, endometrial, hepatocellular, cervical, and gastric cancers, as well as in certain lung tumor subsets, TRIM22 is frequently downregulated or functionally impaired. Where this occurs, its re-expression tends to curb proliferation, invasion, stemness, and cachexia-associated programs. Conversely, in non-small-cell lung carcinoma, glioblastoma, melanoma, and pancreatic ductal adenocarcinoma, TRIM22 often takes on a pro-tumorigenic role. It drives epithelial mesenchymal transition, NF-κB signaling, autophagy, and acquired resistance to chemotherapy or targeted agents. The substrate repertoire of TRIM22 is remarkably broad. It includes p53-related effectors, I-κBα, NF-κB components, β-catenin, TCF4, CCS-STAT3, PHLPP2, Raf-1, p21, ADRB2, CDT2, and Smad2. In this review, we synthesize recent findings and argue that TRIM22 functions as a stress-responsive ubiquitin signaling hub rather than as a conventional oncogene or tumor suppressor. It integrates inputs from interferons, p53, hormonal cues, and inflammatory pathways. The phenotypic outcome, whether tumor suppression or tumor promotion, is dictated by cellular lineage and the pre-existing molecular wiring. Deciphering this contextual logic is the key to translating TRIM22 biology into clinically useful strategies.

## Introduction

TRIM22, also known as Staf50, occupies an unusual biological niche. It sits at the crossroads of antiviral innate immunity, interferon signaling, and tumor pathogenesis. Yet it does not fit neatly into any single category. As an interferon-responsive E3 ligase, it is embedded within broader cellular stress networks that also involve p53 and inflammatory transcription programs. Tumor cells are notorious for hijacking precisely these types of stress-response cascades. That, we suspect, is why TRIM22 has been so consistently polarized in different cancer types. In some settings it clearly limits transformation, while in others it sustains proliferation, survival, stemness, and immune evasion (Lin et al., 2023; Liu et al., 2021; Mohammadi et al., 2022; Pagani et al., 2021).

From an immunological standpoint, TRIM22 is not a cancer gene in the classical sense. It is better understood as a signal transducer that converts immune inputs into cell-fate decisions. Early work pointed to a connection between TRIM22 and p53-dependent growth suppression in leukemia. Altered expression was also noted in Wilms tumor and breast cancer (Obad et al., 2004; Sun et al., 2013; Zirn et al., 2006). Subsequent studies placed TRIM22 among the interferon-stimulated restriction factors involved in viral recognition (Zhang et al., 2004; Si et al., 2006; Hatakeyama, 2011; Kelly et al., 2014; Kelly and Barr, 2014; Forlani et al., 2019). More recently, cancer-focused investigations have extended these immunological foundations. They now implicate TRIM22 in EMT, autophagy, NF-κB, MAPK, STAT3, and stem-cell regulatory circuits. What remains less clear is whether these diverse connections reflect distinct functions of TRIM22 or merely different readouts of a single core activity operating in different cellular contexts.

We begin by examining TRIM22 as a stress-responsive signaling effector modulated by p53, interferons, hormonal signals, and viral oncoproteins. We then survey the experimental and clinical evidence for its tumor-suppressive functions. A separate body of work shows that TRIM22 can drive oncogenesis and therapeutic resistance under different conditions. Finally, we discuss the translational hurdles that complicate biomarker development. We also outline why combination strategies, rather than direct targeting of TRIM22 itself, may offer the most viable path forward. Our underlying premise is that the apparent contradictions surrounding TRIM22 are not artifacts or errors. They are the very essence of its biology (Figure 1).

**FIGURE 1:**
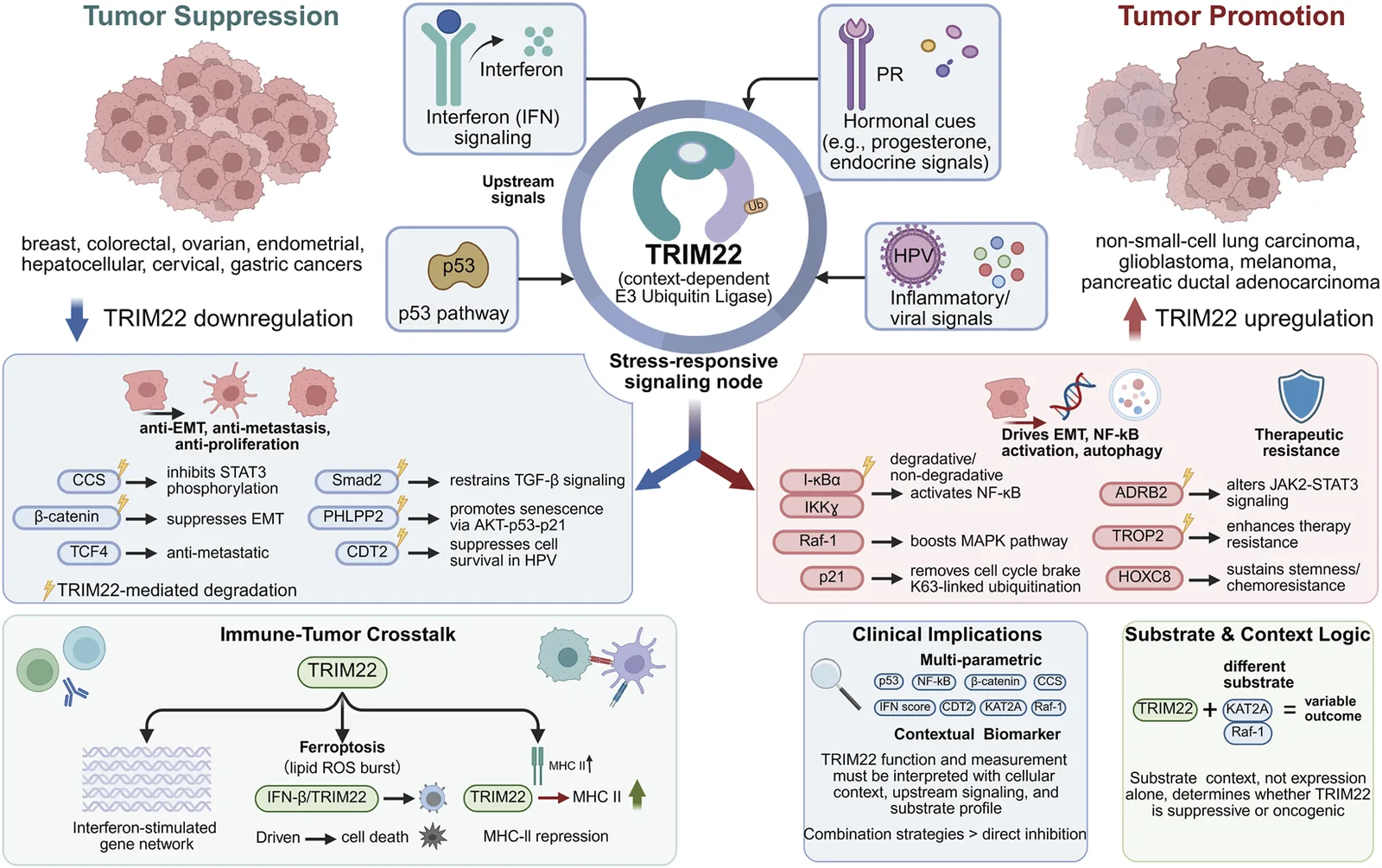
Context-dependent dual roles of TRIM22 in cancer and immune-tumor crosstalk. TRIM22 is a stress-responsive E3 ubiquitin ligase whose biological function is shaped by upstream signaling inputs, cellular context and available substrates. Upstream triggers including interferons, p53, hormonal cues and viral signals drive TRIM22 expression. In breast, colorectal, ovarian and other malignancies, TRIM22 exerts tumor-suppressive effects via degrading oncogenic substrates to restrain proliferation, EMT and metastasis. By contrast, in non-small-cell lung carcinoma, glioblastoma, melanoma and pancreatic ductal adenocarcinoma, TRIM22 promotes oncogenic phenotypes, EMT, NF-κB/MAPK activation and therapeutic resistance through degradative or non-degradative ubiquitination of distinct target proteins. The lower subgraph illustrates TRIM22-mediated immune-tumor crosstalk: TRIM22 participates in interferon-stimulated gene networks, mediates IFN-β-triggered ferroptotic cell death, and represses MHC-II expression. TRIM22 alone is insufficient for clinical interpretation; multi-parametric biomarker profiling combining p53 status, interferon signatures and substrate markers is required for translational applications.

## TRIM22 as a stress-responsive immune gene

It is most appropriate to conceptualize TRIM22 as a context-dependent interferon-inducible E3 ubiquitin ligase that functions downstream of stress signaling rather than as a direct sensor of stress ligands. Its transcriptional output is controlled by interferons, p53, hormonal signals, viral infection, and tissue-specific regulatory programs. Tumors exhibit substantial heterogeneity with respect to p53 mutational status, cytokine milieu, endocrine signaling, and baseline immune pressure. All of these factors shape how TRIM22 behaves. In breast cancer specimens and cell lines, TRIM22 protein abundance is commonly reduced. Notably, the correlation between TRIM22 expression and p53 activity is far more prominent in normal tissues than in malignant counterparts. This finding indicates that p53 loss of function uncouples TRIM22 from its canonical upstream regulatory control (Sun et al., 2013).

In endometrial cancer models, TRIM22 transcription is progesterone-dependent. This finding provides direct evidence that endocrine signaling modulates its abundance within tumor cells (Saito-Kanatani et al., 2015). For HPV-positive cervical cancers, virus-driven p53 degradation lowers TRIM22 levels and permits CDT2 protein accumulation. This observation places TRIM22 within the p53-Rb tumor-suppressive axis rather than operating independently of it (Zhou et al., 2025). Studies investigating bisphenol A exposure in breast epithelial cells further reinforce endocrine-dependent regulation. TRIM22 remains functionally coupled to progesterone receptor transactivation and apoptotic control. These works demonstrate how hormonal microenvironments reshape its oncogenic roles (Ogawa et al., 2023).

Beyond hormone- and p53-centered regulation, TRIM22 is an integral component of interferon-stimulated gene networks. Multiple reports have documented its antiviral restriction activity against HIV, HTLV, Zika virus, and other retroviruses (Zhang et al., 2004; Si et al., 2006; Leal et al., 2018; Charman et al., 2021; Zu et al., 2022). While these investigations are not cancer-oriented *per se*, they offer critical mechanistic background. They explain why TRIM22 becomes frequently activated in interferon-high tumor microenvironments. Malignant cells can co-opt this antiviral transcriptional state. Downstream consequences vary. Interferon-triggered TRIM22 may slow cell proliferation. Alternatively, it can drive EMT, autophagy, and immune escape. Population genetics work on TRIM22 single-nucleotide polymorphisms and adaptive evolution further highlight strong evolutionary selective pressure and structural plasticity. These features make this protein readily repurposed during oncogenic transformation (Kelly et al., 2014; Kelly and Barr, 2014). While these antiviral investigations establish TRIM22’s biochemical capacity as an IFN-inducible E3 ligase, they do not directly demonstrate equivalent functions in human tumors.

## Tumor-suppressive TRIM22 programs

Evidence for TRIM22 tumor-suppressive function emerges from multiple tumor types. Low endogenous TRIM22 correlates with aggressive clinical features. Restoring its expression mitigates malignant phenotypes. Wilms tumor represents one early example. Transcriptomic profiling identified TRIM22 among genes whose altered expression associates with disease progression and poor clinical outcomes (Zirn et al., 2006; Wittmann et al., 2008). In breast cancer patient samples and cell lines, TRIM22 protein levels are frequently diminished. This change arises in part due to disrupted p53-dependent transactivation (Sun et al., 2013). More recent mechanistic work has clarified one key pathway. TRIM22 targets CCS for proteasomal degradation. It thereby dampens STAT3 phosphorylation and limits proliferation and invasiveness (Yang et al., 2024). This work translates correlative observations of TRIM22 downregulation into concrete substrate-level molecular mechanisms. In bladder cancer, O-GlcNAc modification of KAT2A interferes with TRIM22-mediated ubiquitination of KAT2A. This modification allows oncogenic proliferation. These data support the idea that functional TRIM22 acts as a protective barrier against bladder tumor progression (Yang et al., 2026).

Findings from colorectal and ovarian cancer further illustrate TRIM22-dependent tumor suppression. In colorectal cancer, TRIM22 is commonly downregulated. Higher TRIM22 expression correlates with improved prognosis. Mechanistically, it restrains metastatic potential by ubiquitinating β-catenin and suppressing EMT programs (Hu et al., 2025a). Nevertheless, colorectal cancer also exemplifies molecular context-dependent switching. HOXC8 can engage a TRIM22/NF-κB signaling module that sustains stemness and chemoresistance (Tan et al., 2026). Rather than representing contradictory results, this observation reflects how the same ubiquitin node can be rewired to support tumor plasticity under distinct molecular conditions. For ovarian carcinoma, TRIM22 exerts anti-tumor effects by driving ubiquitination and degradation of the pro-metastatic transcription factor TCF4 (Tao et al., 2024). Additional ovarian cancer work connects GATA4, TRIM22, and lysosome signaling axes to metastasis and cachexia-like phenotypes. These studies expand TRIM22 function beyond cell-cycle control to metabolic and microenvironmental remodeling (Yin et al., 2026).

Studies across gastric, endometrial, hepatocellular, and HPV-driven cervical malignancies add further layers of mechanistic detail. In gastric cancer, TRIM22 binds Smad2 and blunts Smad2/3 phosphorylation. It thereby restrains TGF-β-driven migratory and proliferative phenotypes (Zhou et al., 2021). Within endometrial cancer models, TRIM22 represses NOD2-NF-κB signaling to block proliferation, invasion, and cell-cycle progression. It also mitigates IL-6-mediated cachexia-related effects (Zhang et al., 2020; Zhang et al., 2025). In hepatocellular carcinoma, p53-induced TRIM22 targets PHLPP2 and engages AKT-p53-p21 cascades to promote cellular senescence. These findings position TRIM22 as part of stress-induced senescence responses rather than merely a broad anti-proliferative effector (Kang et al., 2024). As noted above, HPV-positive cervical tumors lose TRIM22 following p53 degradation. This loss allows CDT2 accumulation that sustains transformed-cell survival (Zhou et al., 2025). Even in nasopharyngeal carcinoma, TRIM22 features within interferon-associated transcriptional signatures after podoplanin perturbation. This points to its participation in tumor-linked immune remodeling even when it is not the primary oncogenic driver (Hsu et al., 2019).

Collectively, tumor-suppressive phenotypes converge on a shared mechanistic theme. Functional TRIM22 dismantles pro-tumor transcription factors, EMT mediators, and pro-survival proteins. While substrate preferences differ across tumor lineages, this functional logic remains consistent. Its anti-tumor activity cannot be attributed to generic antiviral effects. Instead, biological outcomes rely on specific ubiquitination of lineage-relevant substrate molecules (Mohammadi et al., 2022; Huang et al., 2022).

## Oncogenic TRIM22 programs and therapy resistance

In several cancer types, TRIM22 exerts oncogenic functions and contributes to therapeutic resistance (Table 1). In non-small-cell lung cancer, tumor tissue exhibits elevated TRIM22 levels associated with advanced disease stage, lymph-node metastasis, and inferior survival. It drives proliferation, invasion, and EMT through PI3K/AKT/GSK3β-β-catenin signaling cascades (Liu et al., 2017). A separate investigation described a FOXO3-TRIM22 axis mediating gemcitabine resistance by activating cytoprotective autophagy (Wang et al., 2020). That work highlights that TRIM22 shapes not only baseline tumor growth but also responses to chemotherapy. Conversely, reduced TRIM22 activity in lung adenocarcinoma de-represses ADRB2 and hyperactivates JAK2-STAT3 signaling. These results reinforce that altered TRIM22 balance, rather than simply high or low expression, determines pathway outputs (Xu et al., 2025).

**TABLE 1:** Summary of TRIM22 functions across cancer types.

Tumor type	Direction of TRIM22 change	Substrate/Pathway	Functional outcome	Experimental model	Evidence type
Breast cancer	Downregulated (protein)	CCS → STAT3; p53-dependent transactivation	Tumor suppression: ↓proliferation, ↓invasion	Cell lines, patient tissue	Mechanistic (Yang et al., 2024); Correlative (Sun et al., 2013)
Wilms tumor	Altered expression	Unknown	Associates with disease progression, poor outcome	Transcriptomic patient cohorts	Correlative (Zirn et al., 2006)
Bladder cancer	Functional impairment (KAT2A O-GlcNAcylation)	KAT2A	Tumor suppression when functional: ↓proliferation	Cell lines	Mechanistic (Yang et al., 2026)
Colorectal cancer	Dual: downregulated (suppressive); HOXC8-induced (oncogenic)	β-catenin (suppressive); NF-κB (oncogenic)	Suppression: ↓metastasis; Oncogenic: ↑stemness, ↑chemoresistance	Cell lines, xenografts	Mechanistic (Hu et al., 2025a; Tan et al., 2026)
Ovarian cancer	Downregulated	TCF4; GATA4/lysosome signaling	Tumor suppression: ↓metastasis, ↓cachexia	Cell lines, *in vivo* models	Mechanistic (Tao et al., 2024; Yin et al., 2026)
Gastric cancer	Downregulated	Smad2 → TGF-β/Smad2/3	Tumor suppression: ↓proliferation, ↓migration	Cell lines	Mechanistic (Zhou et al., 2021)
Endometrial cancer	Downregulated	NOD2/NF-κB; progesterone-dependent transcription	Tumor suppression: ↓proliferation, ↓invasion; cachexia protection	Cell lines, *in vivo* models	Mechanistic (Zhang et al., 2020; Zhang et al., 2025)
Hepatocellular carcinoma	p53-induced	PHLPP2 → AKT-p53-p21	Tumor suppression: cellular senescence	Cell lines	Mechanistic (Kang et al., 2024)
Cervical cancer (HPV+)	Downregulated (E6-mediated p53 degradation)	CDT2	Tumor suppression: growth control when p53 intact	Cell lines	Mechanistic (Zhou et al., 2025)
Nasopharyngeal carcinoma	Present in IFN-associated signatures	Unknown	Immune remodeling association	Transcriptomic analysis	Correlative (Hsu et al., 2019)
Non-small cell lung cancer	Upregulated	PI3K/AKT/GSK3β/β-catenin; FOXO3→autophagy	Oncogenic: ↑proliferation, ↑EMT, ↑gemcitabine resistance	Cell lines, patient tissue	Mechanistic (Liu et al., 2017; Wang et al., 2020)
Lung adenocarcinoma	Reduced activity	ADRB2 → JAK2/STAT3	Oncogenic: ↑progression when TRIM22 low	Cell lines	Mechanistic (Xu et al., 2025)
Glioblastoma	Upregulated	I-κBα → NF-κB; Raf-1 → MAPK; IKKγ K63-Ub	Oncogenic: ↑proliferation; immune-inflamed but adverse prognosis	Cell lines, orthotopic xenografts, patient cohorts	Mechanistic (Ji et al., 2021; Fei et al., 2023); Correlative (Hu et al., 2025b; Han et al., 2026)
Melanoma	Upregulated (some datasets report reduced)	p21 (K63 non-degradative Ub); KAT2A/Notch1	Oncogenic: ↑proliferation, cell-cycle progression	Cell lines, patient datasets	Mechanistic (Jin et al., 2025); Correlative (Gu et al., 2023)
Pancreatic ductal adenocarcinoma	IFN-induced (adaptive to KRAS-MAPK inhibition)	I-κBα → NF-κB; TROP2	Oncogenic: ↑EMT, ↑drug resistance (adaptive)	Cell lines, organoid models	Mechanistic (Bulle et al., 2026)
Oral squamous cell carcinoma	Upregulated (PRKCQ-controlled)	Autophagosome-lysosome pathway	Oncogenic: ↑proliferation, ↑metastasis	Cell lines	Mechanistic (Liu et al., 2026)

“Mechanistic” indicates studies demonstrating direct substrate ubiquitination, pathway manipulation, or functional rescue experiments. “Correlative” indicates bioinformatic or expression-profiling associations without direct mechanistic validation. Several tumor types (colorectal cancer, melanoma, lung cancer) exhibit context-dependent dual roles, reflecting the central thesis of this review.

Glioblastoma provides one of the clearest examples of oncogenic TRIM22 activity. TRIM22 accelerates I-κBα turnover and catalyzes K63-linked ubiquitination of IKKγ. These events result in NF-κB hyperactivation and enhanced proliferation both *in vitro* and in orthotopic xenograft models (Ji et al., 2021). Follow-up work reported that TRIM22-dependent degradation of Raf-1 further boosts MAPK signaling to sustain glioblastoma growth (Fei et al., 2023). Multiple bioinformatic analyses of glioma patient cohorts link elevated TRIM22 to immune-activation signatures, poor overall survival, and immune-checkpoint-related gene sets (Hu et al., 2025b; Han et al., 2026). These correlative findings suggest that TRIM22 oncogenic effects in glioblastoma are intertwined with tumor-immune crosstalk and are not purely cell-autonomous.

Studies in melanoma, pancreatic ductal adenocarcinoma, and colorectal cancer stem-cell models further document pro-tumor roles. In melanoma, TRIM22 abundance is increased. It promotes proliferation via K63-linked ubiquitination of p21 (Jin et al., 2025). This observation is mechanistically striking. p21 normally restrains cell-cycle progression. Yet TRIM22 modifies it through non-degradative ubiquitination to remove this growth brake. Upon prolonged pharmacological inhibition of KRAS-MAPK signaling in pancreatic ductal adenocarcinoma, interferon-driven TRIM22-NF-κB signaling emerges as an adaptive response. It promotes EMT and drug resistance. TRIM22 facilitates proteasomal degradation of I-κBα and supports upregulation of TROP2 in this setting (Bulle et al., 2026). As mentioned earlier, HOXC8-TRIM22-NF-κB signaling sustains stemness and chemoresistance in subsets of colorectal tumors (Tan et al., 2026). These findings illustrate how therapeutic or oncogenic pressure rewires TRIM22 into adaptive transcriptional circuits. Oral squamous-cell carcinoma data also connect PRKCQ-controlled autophagy, TRIM22 abundance, and metastatic potential. This situates TRIM22 within broader degradation networks that shape treatment adaptation (Liu et al., 2026).

Post-translational and epigenetic regulatory events can switch TRIM22 function by modulating substrate availability or protein-protein interactions. In bladder cancer, KAT2A O-GlcNAcylation disrupts KAT2A-TRIM22 binding and stabilizes KAT2A protein. This modification amplifies oncogenic transcription programs (Yang et al., 2026). By contrast, melanoma datasets have also reported reduced TRIM22 alongside KAT2A-Notch1 signaling. These results further underscore that substrate context governs whether TRIM22 behaves as suppressor or oncogenic effector (Gu et al., 2023). Notably, a circular RNA transcribed from the TRIM22 genomic locus, hsa_circ_TRIM22, also promotes malignant traits in cervical cancer (Zhao et al., 2024). This reminds us that this gene locus can exert oncogenic effects at RNA levels independent of TRIM22 protein function. Taken together, TRIM22 should not be treated as a uni-directional cancer gene. It represents a context-dependent molecular module integrated into diverse oncogenic signaling networks.

## TRIM22 and the immune-tumor interface

Many cancer-relevant functions of TRIM22 intersect with immune biology. It is important to distinguish between tumor-cell-intrinsic effects that may be triggered by immune signals but occur within tumor cells and direct immune-tumor crosstalk involving antigen presentation, immune-cell recruitment, and T cell recognition.

### Tumor-cell-intrinsic effects intersecting with immune signaling

As an interferon-stimulated gene, TRIM22 bridges innate immune signaling to phenotypic outputs within tumor cells. In Zika virus infection, TRIM22 acts as a functional E3 ligase targeting viral proteins for degradation and limiting viral replication (Zu et al., 2022). This finding confirms its well-established antiviral effector capacity. In infection contexts, TRIM22 is not merely a passive marker of inflammation but an active signaling mediator. Tumor cells can co-opt this same molecular machinery, yet outcomes vary substantially. In pancreatic cancer, for instance, interferon-induced TRIM22 drives EMT and therapeutic resistance under KRAS-MAPK blockade (Bulle et al., 2026). These are tumor-cell-intrinsic phenotypes that may be modulated by immune cytokines but do not by themselves constitute evidence of reciprocal immune crosstalk.

Another immunologically important function relates to antigen presentation. TRIM22 represses MHC-II expression in tumor cells. Accordingly, TRIM22 inhibition elevates MHC-II protein abundance and could theoretically improve CD4-positive T cell recognition of malignant cells (Inoue et al., 2022). Whether this increase translates into more efficient antigen presentation, enhanced tumor-specific CD4^+^ T cell activation, or improved response to checkpoint blockade has not been directly tested and remains an important open question. Functional MHC-II antigen presentation requires coordinated expression of the antigen processing machinery (invariant chain/CD74, HLA-DM), appropriate peptide loading, and co-stimulatory signals. None of which have been evaluated in the context of TRIM22 manipulation. Several transcriptomic studies in hepatocellular carcinoma also associate TRIM22 with immune-cell infiltration and prognostic signatures. These links remain correlative and require direct mechanistic validation (Cao et al., 2022; Zeng et al., 2025).

Ferroptosis represents another critical node connecting interferon signaling, TRIM22, and cancer cell death. IFN-β can trigger ferroptotic cell death in part through TRIM22 upregulation. TRIM22 knockdown dampens lipid ROS accumulation and iron-dependent cell death (Dong et al., 2025). These findings raise the hypothesis that TRIM22-dependent ferroptosis could be exploited in combination with immunotherapy. However, this possibility has not been directly tested. Whether TRIM22-dependent ferroptosis modulates antitumor immune responses in vivo—either by enhancing immunogenic cell death or by promoting immunosuppressive inflammation—requires investigation in immunocompetent preclinical models. It is also notable that ferroptosis can have dual effects: while it may enhance immunogenic cell death in some contexts, it can also induce immunosuppressive microenvironments in others, such as glioma. Similar context-dependent patterns are observed in HCC and colorectal cancer translational datasets. TRIM22 expression tracks with inflammatory status, prognosis, and therapeutic responses rather than enforcing one fixed cellular outcome (Cao et al., 2022; Ma et al., 2026).

### Direct immune-tumor crosstalk

In glioma cohorts, higher TRIM22 expression correlates with immune-infiltration scores and checkpoint-gene signatures (Hu et al., 2025b; Han et al., 2026). These patterns are correlative and imply that TRIM22 may either report on or be associated with inflamed tumor microenvironments; direct mechanistic evidence for TRIM22-mediated immune-cell recruitment or myeloid-cell polarization remains lacking.

TRIM22 participates in retroviral restriction. A polymorphic TRIM22-derived peptide (TRIM22-442C, an arginine-to-cysteine exchange at position 442) serves as a minor histocompatibility antigen presented by HLA-A*0201 and recognized by alloreactive CD8^+^ T cells after allogeneic hematopoietic stem-cell transplantation. This finding reflects TRIM22’s role as a source of alloantigenic peptides rather than a direct regulator of T cell function. TRIM22 has also undergone adaptive evolutionary selection driven by immune pressure (Forlani et al., 2019; Leal et al., 2018; Wolfel et al., 2008; Sawyer et al., 2007). While these antiviral and evolutionary investigations provide mechanistic background, they do not directly establish TRIM22’s functional role in human tumor-immune crosstalk.

## Clinical and therapeutic implications

At present, TRIM22 is likely better suited for biomarker development than as a direct therapeutic target. No universal pan-cancer interpretation can be applied, yet disease-specific patterns are emerging. In breast cancer, low TRIM22 expression or impaired p53-dependent induction signals defective cellular stress responses. By contrast, high TRIM22 accompanied by intact CCS degradation may reflect restrained STAT3 activity (Sun et al., 2013; Yang et al., 2024). Within colorectal cancer, elevated TRIM22 can suppress β-catenin-driven metastasis. Yet HOXC8-driven TRIM22 also fuels NF-κB-dependent stem-cell properties (Hu et al., 2025a; Tan et al., 2026; Ma et al., 2026). Therefore, meaningful interpretation requires parallel measurement of β-catenin, NF-κB activity, HOXC8 expression, and treatment exposure history. For glioblastoma, higher TRIM22 consistently associates with poor prognosis, NF-κB/MAPK activation, and immune-inflamed but clinically adverse tumor states (Ji et al., 2021; Fei et al., 2023; Hu et al., 2025b; Han et al., 2026).

From a therapeutic standpoint, TRIM22 will most likely serve as a contextual marker guiding combination-treatment strategies rather than representing a universal drug target. In tumors where TRIM22 executes tumor-suppressive functions, therapeutic strategies should aim to restore or preserve its activity. In HPV-positive cervical carcinoma, where p53 loss reduces TRIM22 and permits CDT2 buildup. Bladder cancer samples show KAT2A O-GlcNAcylation protects KAT2A from TRIM22-mediated ubiquitination. Breast cancer models demonstrate TRIM22-dependent CCS degradation constrains STAT3 signaling (Zhou et al., 2025; Yang et al., 2024; Yang et al., 2026). Under these clinical scenarios, direct pharmacological inhibition of TRIM22 would prove counter-productive. Conversely, in glioblastoma or pancreatic tumors adapted to KRAS-MAPK inhibition, TRIM22 contributes to NF-κB-driven proliferation, EMT, and drug resistance. These observations make pathways downstream of TRIM22 potential therapeutic vulnerabilities (Ji et al., 2021; Bulle et al., 2026). It is important to note that no specific small-molecule agonists or inhibitors of TRIM22 are currently available for clinical use. All proposed interventions remain pre-clinical.

Interpretation of TRIM22 measurements should always incorporate immune contextual information. Following interferon stimulation, high TRIM22 expression can be beneficial if it promotes ferroptosis or generates antigen-presentation vulnerabilities. Yet it becomes harmful when it drives EMT, autophagy, or stemness. The IFN-β-ferroptosis data demonstrate that TRIM22 can amplify lipid-peroxidation-mediated tumor-cell death. Meanwhile, MHC-II-related work suggests that suppressing TRIM22 may boost tumor-cell MHC-II levels and whether this translates into enhanced CD4-positive T cell engagement requires functional validation in antigen presentation assays and immunocompetent models (Inoue et al., 2022; Dong et al., 2025). These observations are not mutually contradictory. They illustrate that TRIM22 diverges toward distinct biological outputs depending on which downstream signaling branch dominates. Moving forward, TRIM22 should not be measured in isolation but profiled alongside interferon-stimulated gene signatures, MHC-II abundance, ferroptosis markers, and immune-checkpoint readouts.

TRIM22 is repeatedly linked to treatment-adapted cellular states. This highlights challenges for static biomarker assessment. Reported associations include gemcitabine resistance in lung cancer, KRAS-MAPK-inhibitor adaptation in pancreatic cancer, chemoresistant stem-cell phenotypes in colorectal cancer, and doxorubicin-associated adaptive signatures in breast cancer pre-clinical models (Tan et al., 2026; Wang et al., 2020; Bulle et al., 2026; Kumar et al., 2023). TRIM22 may only be induced after kinase inhibition, interferon exposure, DNA damage, or chemotherapy. Therefore, baseline diagnostic biopsies often fail to capture treatment-evoked TRIM22 biology. Longitudinal sampling could help distinguish whether TRIM22 marks reversible adaptive states, stably resistant subclones, or broader interferon-high tumor phenotypes requiring combinatorial intervention.

Finally, TRIM22 may help stratify histologically similar tumors that differ substantially in stress-response and immune wiring. For instance, glioblastoma consistently links high TRIM22 to inferior survival and immune activation. Epithelial malignancies such as breast, gastric, endometrial, and ovarian cancer more frequently manifest tumor-suppressive TRIM22 functions (Tao et al., 2024; Zhou et al., 2021; Zhang et al., 2025; Hu et al., 2025b; Han et al., 2026). Future biomarker panels could integrate TRIM22 expression together with p53 status, interferon-response scores, NF-κB activity, β-catenin localization, and substrate-specific markers such as CCS, p21, CDT2, KAT2A, or Raf-1. Such multi-parameter readouts would better reflect underlying biology compared with simple binary classification of TRIM22 as high versus low.

## Discussion

What then is the biological role of TRIM22 within cancer. The most reasonable conclusion is that TRIM22 acts as a context-dependent E3 ubiquitin ligase. Its functional output is dictated by upstream stress inputs plus the spectrum of accessible substrates within a given cell. When p53 signaling remains intact and tumor-suppressive substrates are present, TRIM22 predominantly restrains oncogenic progression. However, under inflammatory signaling, following KRAS-MAPK inhibition, or within lineage-specific rewired networks, substrates such as I-κBα, p21, Raf-1, or ADRB2 become available. Under these conditions, TRIM22 reinforces EMT, autophagy, stem-cell properties, or immune-adapted phenotypes. This apparent duality is not experimental contradiction. It represents the core biology of this molecule (Liu et al., 2021; Mohammadi et al., 2022; Huang et al., 2022).

From a translational perspective, TRIM22 cannot be treated as a universal oncogene or tumor-suppressor biomarker. Its clinical utility depends heavily on tumor type, mutational background, interferon activity, hormonal milieu, and the transcriptional state of the tumor. In p53-proficient tumors with active interferon signaling, TRIM22 may reflect activated defensive stress responses. In tumors driven by KRAS or constitutive NF-κB signaling, identical TRIM22 expression levels may signal adaptive therapeutic resistance. When evaluating TRIM22 in immunocompetent experimental systems, one must jointly assess immune infiltration, MHC-II levels, ferroptosis, and cytokine programs rather than relying solely on TRIM22 abundance as a standalone marker (Mohammadi et al., 2022; Huang et al., 2022; D'Amico et al., 2021).

Three major knowledge gaps should guide future experimental design. First, comprehensive substrate profiling across diverse tumor lineages is essential. The same E3 ligase engages distinct target sets depending on cellular state, and substrate context is what ultimately determines phenotypic outcome. Second, mechanistic studies must make greater use of immunocompetent pre-clinical models. These will clarify under which conditions TRIM22 enhances antigen presentation or ferroptotic tumor-cell death versus promoting treatment resistance. Third, translational research needs to evaluate whether TRIM22 should be directly targeted pharmacologically or if it is more valuable as a companion biomarker for interferon-based, KRAS-targeted, or immunotherapy regimens. The field has clearly established that TRIM22 matters in cancer. The main challenge moving forward is defining the precise circumstances where it confers protection versus driving disease and uncovering molecular switches that toggle between these opposing functions.
